# Intravenous or subcutaneous natalizumab in patients with relapsing–remitting multiple sclerosis: investigation on efficiency and savings—the EASIER study

**DOI:** 10.1007/s00415-023-11955-0

**Published:** 2023-09-16

**Authors:** Massimo Filippi, Luigi Grimaldi, Antonella Conte, Rocco Totaro, Maria Rosaria Valente, Simona Malucchi, Franco Granella, Cinzia Cordioli, Vincenzo Brescia Morra, Chiara Zanetta, Daria Perini, Laura Santoni, the EASIER Study Working Group, V. Ziccone, R. Garba, A. Motta, S. Albanesi, O. Oddo, A. Impagliato, G. Ferrazzano, V. Di Rosa, M. Tartaglia, A. Di Vito, A. Galassi, R. Prota, R. Garbo, I. Del Negro, L. Nesi, M. Capobianco, E. Tsantes, R. Agosta, A. M. Valleri, L. Galuppini, S. Mascara, F. Bertocchi, E. Chiarini, M. Moccia, G. Puorro

**Affiliations:** 1 Neurology Unit, Neurorehabilitation Unit, Neurophysiology Service, and Neuroimaging Research Unit, Division of Neuroscience, IRCCS San Raffaele Scientific Institute, Via Olgettina, 60, 20132 Milan, Italy; 2 Vita-Salute San Raffaele University, Milan, Italy; 3 Multiple Sclerosis Center, Institute Foundation G. Giglio, Cefalù, PA Italy; 4 Department of Human Neurosciences, Sapienza University of Rome, Rome, Italy; 5 Multiple Sclerosis Center, Policlinico Umberto I Hospital, Rome, Italy; 6 IRCCS Neuromed, Pozzilli, IS Italy; 7 Demyelinating Disease Center, Department of Neurology, San Salvatore Hospital, L’Aquila, Italy; 8 Clinical Neurology, Santa Maria della Misericordia University Hospital, and Department of Medicine, University of Udine, Udine, Italy; 9 SCDO Neurologia, S. Luigi Gonzaga University Hospital, Orbassano, TO Italy; 10 Department of Medicine and Surgery, University Hospital of Parma, Parma, Italy; 11 Multiple Sclerosis Center, ASST Spedali Civili di Brescia, Montichiari Hospital (Brescia), Brescia, Italy; 12 Multiple Sclerosis Clinical Care and Research Center, Department of Neuroscience (NSRO), Federico II University Hospital, Naples, Italy; 13 Biogen Italia, Milan, Italy

**Keywords:** Natalizumab, Multiple sclerosis, Intravenous administration, Subcutaneous administration, Costs, Time

## Abstract

**Introduction:**

EASIER is a multicenter, observational, cross-sectional study investigating the consumption of healthcare resources, including healthcare professional (HCP) active working time, the costs associated with the current natalizumab intravenous (IV) administration, and the potential impact of the adoption of subcutaneous (SC) route.

**Methods:**

The EASIER study has three parts: (1) time and motion study to measure healthcare resources and working time needed for natalizumab IV administration using a digital data collection tool operated directly by HCPs; (2) HCP structured questionnaire-based estimation of the potential impact of natalizumab SC vs. IV administration; and (3) patient survey on the burden of natalizumab administration.

**Results:**

Nine Italian multiple sclerosis (MS) centers measured 404 IV natalizumab administration procedures and administered 26 HCP questionnaires and 297 patient questionnaires. Patients had a mean of 52 (range 1–176) previous IV administrations and spent a mean (median, IQR) of 152 (130, 94–184) minutes in the center per each IV procedure, with IV infusion covering 50% of the total. Including patient travel time, an average of 5 h was dedicated to each IV administration. Active working time by HCP amounted to 29 min per IV administration procedure, 70% of which by nursing staff.

With adoption of the SC route, HCPs estimated a 50% reduction in patient procedure time and 55% lower HCP active working time. This translated into a 63% cost reduction for the MS center per natalizumab administration procedure.

**Conclusions:**

SC natalizumab administration will consistently reduce consumption of patient and HCP times per procedure and associated costs.

**Supplementary Information:**

The online version contains supplementary material available at 10.1007/s00415-023-11955-0.

## Introduction

Multiple sclerosis (MS) is an inflammatory disorder affecting the central nervous system and causing several clinical deficits. It is the most common neurological cause of disability in young adults [1]. The most recent analyses from the World Health Organization estimate a global prevalence of 2.8 million people [1], i.e., 36:100,000 persons, but with different distribution among countries, with Europe reporting the greatest prevalence (133:100,000) and Western Pacific and Africa the lowest one, i.e., 5:100,000 (likely to be influenced by lack of data). In Italy, the current total number of patients with MS diagnosis is estimated to be around 127,000 with a prevalence of 208:100,000 [2]. Each year, 3400 Italian citizens are diagnosed with MS (incidence = 5.5:100,000) [2], mainly females (69%), mean age at diagnosis 32 years [1]. All over the world, the number of affected people is growing, as a result of improved diagnosis, improved counting methods, increased survival (due to more effective treatments), and growing population [1].

The total cost of MS in Europe in 2010 was estimated at € 14.6 billion [3]. The overall cost of the disease in Italy was € 4.8 billion and the mean annual cost per patient was estimated at € 39,307 in 2019 [4].

Natalizumab (Tysabri^®^, Biogen, Cambridge (MA), USA) is a recombinant humanized monoclonal antibody targeting α4-integrin on the surface of leukocytes, approved as an MS disease-modifying therapy (DMT). Through its mechanism of action, it prevents leukocytes from crossing the blood–brain barrier, thereby suppressing inflammatory activity at the disease site and inhibiting further recruitment of immune cells into inflamed tissues [5]. It has long been indicated as an intravenous (IV) infusion in adults with highly active relapsing-remitting MS (RRMS) despite a full and adequate course of treatment with at least one DMT or with rapidly evolving severe RRMS [6].

In the phase III pivotal clinical trial AFFIRM [7], 942 patients affected by RRMS were recruited and randomized to receive natalizumab 300 mg or placebo IV every 4 weeks for more than 2 years. Natalizumab reduced the risk of sustained progression of disability by 42%, the rate of clinical relapse at one year by 68%, and the accumulation of new or enlarging T2-hyperintense lesions by 83%. Its effectiveness has been confirmed in several real-world studies, which demonstrated: increased proportion of patients reaching the No Evidence of Disease Activity (NEDA) status [8], reduced Expanded Disability Status Scale (EDSS) score [9], reduced cortical lesion accumulation and cortical atrophy progression [9–12], improvement in cognitive function [12–16], quality of life [14, 17], fatigue [14, 18], and productivity [19–21].

In 2021, the European Commission authorized the formulation for subcutaneous (SC) administration of natalizumab [5]. Two studies compared the IV and the SC administrations of natalizumab: DELIVER [22] and REFINE [23] studies.

The DELIVER study found that mean serum concentrations after repeated dosing were similar with SC or IV administration. Pharmacodynamic characteristics, safety, tolerability, and immunogenicity were comparable among all the administration routes considered.

Similarly, the REFINE study proved that natalizumab 300 mg administered SC every 4 weeks was comparable to the IV administration with the same dosage and frequency regarding efficacy, pharmacokinetics/pharmacodynamics, and safety.

The advantages of the SC formulation over the IV formulation at the same dosing frequency have been extensively proved in other therapeutic areas, thus providing a rationale to support the value of SC natalizumab. These experiences suggest that the SC formulation can potentially better meet patients’ needs and improve the efficiency of Healthcare Services.

In particular, studies on other monoclonal antibodies, such as trastuzumab and rituximab, showed that SC administration with respect to IV administration:

- facilitates the management of administrations [24];
- greatly reduces the infusion chair time [25, 26];
- reduces the healthcare professional’s (HCP) time [25, 26];
- increases the number of treatable patients, thus reducing the waiting list [25];
- decreases the time spent by patients in hospital [27];
- reduces the direct healthcare costs for the National Healthcare Service, as it significantly decreases the use of consumables (syringes, cotton, infusion set, flow regulator, etc.) [24, 28, 29];
- decreases the indirect costs (loss of productivity) [28, 30];
- in most cases is the preferred route of administration for the patients [28];
- improves patients’ quality of life (QoL) as a result of increased convenience associated with shorter administration times and increased flexibility [24];
- eliminates the need for venous access and allows the use of more injection sites [29].

This study aimed at evaluating the economic impact associated with the use of natalizumab SC vs. natalizumab IV in patients with RRMS from the perspectives of the MS center, the patient, and the society.

## Methods

### Study design

EASIER is a multicenter, observational, cross-sectional study carried out from July 19, 2021 to November 30, 2021 in 9 Italian MS centers.

Specifically, the EASIER study consists of three parts:

- part 1: measurement of administration time, HCPs working time and healthcare resource consumption associated with the IV administration of natalizumab conducted by means of an ad hoc App (EASIER App, see below), operated directly by HCPs involved in the IV natalizumab administration (time & motion methodology);
- part 2: analytic parametric estimation of administration time, working time of HCPs, and healthcare resource consumption expected for the SC administration of natalizumab, by means of a structured questionnaire administered to the HCPs involved in part 1;
- part 3: estimation of direct, indirect, and intangible costs and QoL for patients receiving IV natalizumab and expected impact of the shift to the SC administration, by means of a structured questionnaire administered to the treated MS patients.

### Participating centers

The participating centers are listed here: IRCCS San Raffaele Scientific Institute (Milan), Fondazione Istituto G. Giglio (Cefalù, PA), University Hospital Policlinico Umberto I (Rome), San Salvatore Hospital (L’Aquila), Santa Maria della Misericordia University Hospital (Udine), S. Luigi Gonzaga University Hospital (Orbassano, TO), University Hospital of Parma (Parma), Montichiari Hospital (Brescia), and Federico II University Hospital (Naples).

### Population

The EASIER study recruited adult RRMS patients presenting in any of the participating centers to receive their usual IV natalizumab administration, according to standard clinical practice. Inclusion criteria were the following: age ≥ 18 years old, RRMS diagnosis according to McDonald criteria, treatment with IV natalizumab according to an approved indication, access to the clinical center due to a previously scheduled natalizumab administration, and willingness to participate in the study by signing the informed consent form.

### Endpoints

The primary endpoint was the comparison of the mean cost of IV and SC administration routes of natalizumab in patients affected by RRMS from the MS center perspective. The following costs were considered: healthcare resources consumed (medications and consumables), active working time spent by the HCPs, and use of durable equipment.

The secondary endpoints were:

- the evaluation of the active working time of HCPs (total time and time per every role of HCP and per each task)—HCP time;
- the evaluation of patient’s length of stay in the MS center for the IV administration—patient total time;
- the evaluation of time of durable equipment use (infusion chair or bed)—chair total time;
- the evaluation of the mean cost for IV vs. SC administration routes of natalizumab from the patients and society perspectives—total costs and costs per every component, direct non-healthcare costs (such as transportation and formal non-healthcare), indirect costs (such as loss of patient productivity), and intangible costs (such as decreased quality of life perceived by the patient).

### Data collection

Patients were proposed to participate in the study during a previously planned access to the MS center for their usual IV natalizumab administration: clinicians or nurses explained the design and the purpose of the study, provided patients with written information, and collected the informed consent authorizing the process of personal data of patients willing to participate in the study.

In part 1, every participating center was asked to measure time and resource consumption of a number of natalizumab IV infusion procedures (≤ 50 infusions) in the context of their routine clinical practice. A protocol amendment allowed centers to recruit a higher number of patients, thus 3 centers exceeded 50 patient recruitments. HCPs performed the measurements through the EASIER App, which allowed the selection of a single procedure and a specific task to be measured during the natalizumab IV administration. Every single measurement occurred using the start/stop buttons. Resources used for the procedure were associated directly through a selection of pre-defined items. The App allowed for concurrent use by several HCPs along with the measurement of concomitant procedures, by a single or more operators. The EASIER App was installed on the mobile devices of the participating researchers.

The estimation of administration time and resource consumption of natalizumab SC and of the potential impact of SC administration (part 2) was performed by HCPs of participating MS centers through an ad hoc prepared questionnaire called “How much EASIER?”, which was provided online or in printed form (if specifically requested). Data were recorded in the EASIER study centralized database. Printed forms were scanned and sent to be manually entered into the database. Each center was asked to enroll at least one person per every specific professional role (i.e., one physician, one nurse, etc.).

The estimation of costs and QoL of patients under IV natalizumab and of the potential impact of SC administration (part 3) was collected through an ad hoc prepared questionnaire called “EASIER for you?”, which was provided online or in printed form (if specifically requested by the patients) and was filled by enrolled patients themselves or with the help of a caregiver or an HCP. All collected data were organized in the centralized database of the EASIER study. In the questionnaire, patients also had to evaluate their QoL by means of the Visual Analog Scale (VAS) scale, ranging from 0 (the lowest possible QoL) to 100 (the highest possible QoL). Additional explorative analyses were carried out on the preferences the patients assigned to the proposed routes of administration.

No follow-up was planned, as data were collected just once, according to the cross-sectional design of the study.

The sample size, thus, obtained was deemed to be appropriate to carry out a health economics analysis.

Data collection for the EASIER study was planned to end on November 30, 2021.

### Data management

To ease the measurements by the EASIER App, the administration procedure was split into macro-tasks, as reported in Online Resource 1. This also shows how such timings were aggregated in the subsequent analyses.

When data were lacking due to errors in the recording of execution times, the mean value of the lacking task estimated in the same center was inserted. The execution times for the SC administration were calculated by applying to the IV elaborated data the possible mean absolute difference estimated by HCPs who answered to the relevant questions in the survey “How much EASIER?”.

### Statistical analyses

The categorical variables are represented in terms of absolute and relative frequencies (percentages), whilst the continuous variables are synthesized by showing mean, standard deviation and, if deemed informative, total range (min–max).

To account for the possible differences concerning the organization among the enrolled centers, total costs and times of intravenous procedures (HCP time and patient time) were also estimated through a random-intercept regression model.

The variables were adjusted according to the number of previous infusions of each enrolled subject, to take into account for the possible confounding effect of HCP experience in managing each single patient (i.e., the execution times may be longer in new patients as HCP may have to explain some steps or answer patients’ questions).

Briefly, the model used in this analysis is shaped as follows:

$$Y={\alpha }_{\mathrm{center}}+\beta \times \#\mathrm{infusions} +\varepsilon ,$$

where *Y* is the dependent variable to be analyzed (cost/HCP and patient time), *α*_center_ represents the estimation of *Y* in the base case (i.e., a patient without previous infusions), *β* describes the increase in *Y* value for every previous additional infusion, and *ε* represents the error term on the observed data (assumed as normal with mean = 0 and the same variance for every measurement). In the random-intercept model, *α*_center_ parameter follows a normal distribution centered on a mean value *α* with variance *τ*_center_, which describes the variability in the measure *Y* for every enrolled center.

Non-parametric Wilcoxon test was used to evaluate the difference between groups. The impact of the employment status and the previous experience with other SC therapies was further investigated by multivariate regression analysis that evaluated the association between the measured factors and the preference expressed by the patients about the alternative administration routes, whilst at the same time adjusting for the QoL in the days without administrations and other possible confounders. These analyses were of an incremental nature, i.e., they did not focus on the absolute values assigned by the patients to the various modalities proposed, but on the difference in scores assigned to mode pairs: as a result, the analyzed sample did not correspond to the total, but included only the patients who assigned a value to the analyzed modalities.

### Health economics analyses

#### MS center perspective

A pertinent unit cost was assigned to every resource consumed. Unit costs were collected by the App or derived from the national literature.

The cost of the working time of HCPs was estimated according to the opportunity-cost principle and set at the mean national values reported by the Italian National Institute of Statistics (ISTAT), i.e., 67 €/h, 27 €/h, 25 €/h, and 25 €/h for physicians, nurses, unlicensed assistive personnel (UAP), and administrative personnel, respectively [31].

The depreciation cost, which was assigned to the use of durable equipment, was estimated assuming a 10-year duration with a use equal to 8 hours a day and 260 working days a year [32]. Basing on the article from Schivazappa et al. [32], the infusion chair and the IV pole were assigned values equal to 3,400 € and 100 €, respectively.

The consumption of the materials needed to administer the drug was estimated using the unit costs collected during the study.

In accordance to the good activity-based accounting practice [33], hospital general costs, which included cost items that are non-specific to the department where the administration took place, must also be considered when evaluating the total cost. Hospital general costs were reversed pro quota on the center according to the activity provided and included: intermediate healthcare services, common costs, general and administrative costs, depreciation, purchases of non-healthcare goods and services. To this end, 25% was added to the intermediate calculation thus obtained, based on the general estimate that hospital general costs represent 20% of the total costs of a healthcare service [33].

#### Patient and society perspective

The cost areas investigated in the survey entitled “EASIER for you?” included non-healthcare and indirect costs. In the first case, resources that patients themselves had to pay to get access to the therapy (e.g., transport costs to the MS center) were considered. Indirect costs are the economic value of the productivity lost by the patient due to his/her reduced functionality or by the caregiver to provide informal assistance to the patient.

In particular, the following non-healthcare expenses borne by the patient were investigated: the costs concerning the transport from home to the MS center and the formal assistance for the patient (accompanying to the MS center), his/her relatives (e.g., baby-sitter), or home care (e.g., maid) for the period in which the patient is at the center.

Transportation costs were estimated based on reported distance and type of transportation, valued according to public official sources [34–36]. For travel by plane, a flat rate for round-trip flights in Italy of 100 € has been assumed.

The direct costs paid by the patient for a caretaker/accompanying person or for a maid/baby-sitter was calculated on the basis of the national collective agreement according to level and training [37].

The hours in which patients were unable to perform their daily duties because they had to travel to the MS center to receive IV therapy were translated into indirect costs according to the following scheme:

- the mean number of working hours lost (absenteeism, time-off requests) was divided into:
  - hours paid by social security bodies, which represent an indirect cost for society and were valued, according to Human Capital Theory, through the gross hourly wage [38] (updated to 2021);
  - hours not paid by social security bodies (lost earnings), which represent an indirect cost suffered firstly by the individual patient, and secondly by the society. Accordingly, it was reasonable to value it through the hourly wage net of the tax burden (mean tax burden = 25%).
- the number of hours “stolen” from one’s routine unpaid tasks is also an indirect cost for the society and have been valued using the Market Cost Method (evaluation of unpaid tasks at the market price of a substitute’s work). The time that each person, depending on age group and gender, dedicates to housework, to the care of cohabiting family members or other families, and to volunteering was quantified through the ISTAT data collected in “L’uso del tempo” report [38]. These activities have been monetized through the minimum wages of housekeepers (super B level of the CCNL, 2021 [37]), caretakers (mean between super B level for autonomous patients and super C/D for non-autonomous patients [37]), and indices of the CNEL-ISTAT report for the economic evaluation of voluntary work [39] (updated to 2021), respectively. These costs were spread over 16 h to obtain a mean cost per hour of a routine day (counting 8 fixed and “non-usable” hours of sleep/rest). The abovementioned costs per hour are shown in Online Resource 2.

The time dedicated by informal caregivers to assist and go with the patient to the MS center where he/she receives IV therapy was valued according to the following scheme:

- the mean number of hours used multiplied by the hourly wage of a caretaker (mean between super B level for autonomous patients and super C/D for non-autonomous patients [37]) according to the Human Capital Theory, if the caregiver had no paid employment;
- the mean number of hours used multiplied by the value that time would have had if it had been dedicated to one’s usual job [38] (updated to 2021) if the caregiver had a paid job.

The abovementioned costs per hour are also shown in Online Resource 2.

## Results

### Part 1: time and resources for IV administration

Among the 9 MS centers involved, 404 natalizumab intravenous administration procedures were evaluated.

The number of procedures evaluated per center ranged from 15 to 80. Patients had undergone on average 52 previous administrations (median = 43, interquartile range—IQR = 18–85, range = 1–176), as reported by HCPs who carried out the measurement.

Table 1 shows that patients spent on average 2.5 hours (152 minutes) in the MS center to receive the intravenous administration of natalizumab and about half of the time was dedicated to the actual infusion.

**Table 1:** Patient and HCP time spent for the intravenous administration

	Patient time (min)		HCP time (min)	
	Mean (SD)	Proportion (%)	Mean (SD)	Proportion (%)
Pre-infusion^a^	18 (57)	12	14 (17)	47
Infusion	77 (29)	50	3 (6)	11
Post-infusion^b^	58 (47)	38	13 (16)	43
Total	152 (79)	100	29 (16)	100

In some cases, rounding prevents the sum to seem precise

*HCP* healthcare professionals, *SD* standard deviation

^a^Including tasks 1–4 of Online Resource 1

^b^Including tasks 6–8 of Online Resource 1

Overall, median total time was equal to 130 min, IQR = 94–184 min. The mean time per patient varied among centers from 86 to 213 min. The analysis using the random-intercept linear regression model showed a mean time equal to 143 min (95% confidence interval—95% CI 119–167 min). Online Resource 3 showed a statistically significant center effect, whilst previous infusions had no effect on the mean time per patient.

On average, HCPs devoted approximately half an hour (29 min) of active working time to intravenously administer natalizumab, with about half the time dedicated to the preparation before the actual infusion (Table 1).

Globally, median working time by the HCP was 23 min, IQR = 10–31 min. The analysis using the random-intercept linear regression model estimated a mean working time of 28 min (95% CI 22–34). As to patient time, the center effect was statistically significant and the number of previous infusions seemed to have no effect on the mean time per HCP, as reported in Online Resource 4.

Concerning the time spent by HCPs, the prominent role was played by the nurse, whose time summed up to 70% (21 min) of the total time. The total remaining time was distributed as follows: physician 27% (corresponding to 8 min) and UAPs and administrative personnel 2% (0.5 min) and 1% (0.3 min), respectively.

Healthcare resources consumed during the administration of intravenous natalizumab were also measured: average utilization varies only slightly between centers (see also the “Economic impact analysis” paragraph below). The infusion chair/bed was occupied for an average of 125 (± 62) minutes per procedure.

### Part 2: how much EASIER? questionnaire. Time and resources with subcutaneous administration

The answers of 10 nurses, 13 neurologists, and 3 administrative employees (trial coordinators) to the How much EASIER? questionnaire were collected. Only 5 HCPs among responders had experience with SC natalizumab administration.

Responders were asked to consider each task that was measured for IV administration and estimate a decrease, increase, or maintenance of the time if the administrations were subcutaneous (Online Resource 5).

Considering the task organization shown in Online Resource 1 and the times for measurements recorded for IV administrations (Table 1), and applying the expected time reductions according to the respondents to the survey “How much EASIER?” (Online Resource 5), the estimated times for SC administrations were obtained (Table 2).

**Table 2:** Comparison between measured times for IV and estimated times for SC administration of natalizumab

Times	Measured IV time (min)	Estimated difference (min)	Estimated SC time (min)
Total patient time	152	− 73	78
Total infusion chair time	125	− 74	51
Total HCP time	29	− 16	13
Pre-infusion	14	− 8	6
Infusion	3	− 2	1
Post-infusion	13	− 6	6

In some cases, rounding prevents the sum to seem precise

*HCP* healthcare professionals

Overall, we estimated that the total patient stay in the center was 78 min for each subcutaneous administration of natalizumab, thus depicting almost a 50%-time reduction in comparison to the current IV administration procedure. Infusion chair time was also estimated to decrease in a consistent manner (− 74 min, i.e., − 59%). Concerning HCP time, only 13 min were considered to be spent for SC administration, in comparison with 29 calculated for IV administration (− 55%).

HCPs were also asked their opinion about the new administration route. No one was against the use of SC administration, whilst 92% were in favor or very much in favor of it.

### Part 3: EASIER for you? questionnaire. Impact on the patient

The responders of the questionnaire “EASIER for you?” were mainly female (69%), had a mean age of 37 years, and had been in treatment with natalizumab for approximately 4 years (Table 3).

**Table 3:** Data from the responders of the survey “EASIER for you?”

	N. (%) responders	Mean (SD)	Range
Patients data			
Patients	297 (100%)		
Age	293 (99%)	37 (10)	19–65
N. male (age)	92 (31%)	37	21–65
N. female (age)	205 (69%)	38	19–60
Time in treatment with natalizumab (months)	294 (99%)	49 (35)	1–176
Previous treatments			
None	159 (54%)		
Any (n. treatments)	138 (46%)	1.4 (0.6)	1–3
Any (months in treatment)	138 (46%)	51 (50)	1–290
Oral drugs (months)	96 (32%)	23 (28)	0–120
Subcutaneous drugs (months)	125 (42%)	35 (41)	0–208
Intramuscular drugs (months)	72 (24%)	31 (41)	0–208
Intravenous drugs other than natalizumab (months)	40 (13%)	18 (34)	0–170
Impact of IV natalizumab on everyday life			
Hours per administration	293 (99%)	5.6 (2.9)	0–25
Among those who have a paid job	210 (71%)	5.9 (2.8)	1–25
Paid job (responder)	294 (99%)		
Paid job (yes)	211 (72%)		
Working hours lost due to administration (among those who have a paid job)	200 (95%)	5.2 (2.8)	0–25
Hours reimbursed	167 (84%)	71% (44%)	0–100%
Caregiver accompanying the patient			
Caregiver (responder)	286 (96%)		
Caregivers (yes)	138 (48%)	1.4 (0.7)	1–4
Age		51.7 (13.5)	20–80
Sex (male)	107 (58%)		
Role			
Nurse or caretaker	0 (0%)		
Informal	166 (100%)		
Of whom with paid job	41 (35%)		

*SD* standard deviation

Around half of the respondents (54%) took natalizumab as first-line therapy for RRMS (naïve patients).

Those who had a paid job (more than 70%) declared that they lost around 5 working hours per administration, 71% of which were reimbursed thanks to welfare measures (Table 3). Furthermore, 10% of patients had to stop working due to their absences from work to receive natalizumab administration, 64% of whom found a new job subsequently. For the same reason, 21% and 7% of respondents reduced their working hours in a self- or company-imposed manner, respectively. A further 11% reduced the level of their duties. Overall, one-third of the patients found that the need for periodic absences to receive the therapy negatively affected their working life.

The round trip to the MS center added up an average distance of 73.6 km, covered mainly by private vehicle (86% of respondents) in an average time of 1.6 h (Online Resource 6). There was great variability in the distance between the MS center and patients’ home (range = 0.75–1200 km) and how long it took to reach it (range = 0–5 h).

About a half of patients went to the MS center with an informal caregiver, who, in 58% of cases, was a male. The mean age (SD) of caregivers was 51.7 years (± 13.5 years). No respondents (*n* = 286) were accompanied by a nurse or a formal caretaker, while friends or relatives were always involved. More than one-third of informal caregivers had a paid job. On average, 1.4 accompanying people were needed per patient and participated in 65% of transfers; 39% of patients declared they always needed an informal caregiver.

The absence from work of the patient (and possibly his/her caregiver) due to natalizumab infusions resulted in the need for paid personnel in 9% of cases (Online Resource 7). One to 3 paid professionals (mean = 1.2 ± 0.5) were needed, who were baby-sitters in 46% of cases (Online Resource 7). They were required 70% of times for 4.6 h on average.

### Quality of life

As expected, in the days on which they had to go to the MS center, patients perceived a lower QoL (66 ± 23) with respect to standard days (79 ± 19). A lower impact on the QoL was expected in case of subcutaneous administration (69 ± 23).

Employment status was strongly and positively associated with the value assigned to a possible SC natalizumab administration (*p* value = 0.0104). Conversely, the previous experience with other SC therapies showed no association with the value assigned to a possible SC natalizumab administration (Online Resource 8).

When analyzing the overall mean differences among the preferences that patients assigned, a delta equal to − 12.2 was found for days without administration vs. days with IV administration. This delta decreased when the comparison was between days with no administration and days with SC administration (− 8.4). A direct comparison of days with IV and SC administration was in favor of the latter (+ 2.3).

The multivariate analysis showed a significant effect of the employment status for SC administration. The experience with other SC therapies showed no significant effects.

### Economic impact analysis

#### Mean cost per administration procedure from the perspective of the MS center

The material consumption for the IV and SC administrations and the relevant economic valorization are reported in Table 4.

**Table 4:** Material consumption and economic valorization

Material	Unit cost (€)	IV natalizumab		SC natalizumab	
		Consumption (measured)	Cost (€)	Consumption (estimated)	Cost (€)
Band-aid	0.19	1	0.19	2	0.38
Disposable gloves	0.29	6	1.71	2	0.57
100 ml saline solution	0.29	3	0.88	0	–
Gauze compresses	0.015	5	0.08	5	0.08
Disinfectant	0.93	0.02	0.02	0.04	0.04
Peripheral intravenous catheter/Huber/butterfly	0.33	1	0.33	0	–
Syringe with needle	0.20	1	0.20	0	–
Detergent	2.39	0.02	0.05	0.02	0.05
Infusion connection	1.07	1	1.07	0	–
IV administration set	1.41	1	1.41	0	–
Peripheral venous catheter dressing	0.29	1	0.29	0	–
Kidney basins	0.05	2	0.10	1	0.05
Film	0.51	1	0.51	1	0.51
Tourniquet	0.15	1	0.15	0	–
Total materials			6.82		1.67

In some cases, rounding prevents the sum to seem precise

Table 5 shows the estimates of direct healthcare costs borne by the MS center for each hospital administration procedure of natalizumab, according to the administration route.

**Table 5:** Mean cost per procedure from the perspective of the MS center

Cost item	IV procedure			SC procedure			Delta (€)
	Use (min)	Unit cost^a^ (€)	Cost (€)	Use (min)	Unit cost^a^ (€)	Cost (€)	
HCP time	29	38	18.63	13	35	7.66	− 10.98
Chair time	125	0.17	0.69	51	0.17	0.28	− 0.41
Materials^b^			6.82			1.67	− 5.16
Hospital costs			6.45			2.37	− 4.08
Total			32.67			12.00	− 20.67

In some cases, rounding prevents the sum to seem precise

^a^The unit cost of HCP time was obtained as the weighted average of national average wages, as reported by ISTAT in 2018 [31]; it was different in the two procedures due to the different composition, with greater relative relevance of the physician’s time in the IV procedure with respect to SC procedure.

^b^Details in Table 4

The mean total cost per IV procedure ranged between € 23 and € 33 depending on the center with a mean cost of € 32.26. The random-intercept linear regression model estimated a mean cost per IV administration of € 30 (95% CI 25–35), unaffected by the number of past infusions (− 0.003, 95% CI − 0.03 to 0.02), but with a significant effect of the center, depending on the impact of the latter on the patient’s total working and stay time (Online Resources 3 and 4). The uncertainty on the total cost did not affect the overall saving estimate that could be obtained by adopting the SC route of administration.

The expected total cost reduction if natalizumab was administered subcutaneously rather than intravenously was approximately € 20 for every administration. More than 50% of this reduction was due to the decrease in the active working time spent by HCPs to administer the drug. The saving was due also to the decrease in the consumption of healthcare materials and the use of equipment, as well as in the shorter stay at the MS center. This was a reduction of about two-thirds from the estimated total cost of IV administration (about € 32 per session). However, it should be noted that the absolute value of the difference was not sensitive to the estimate of the cost per IV administration, as it was based on the reductions in the consumption of direct healthcare resources estimated on the basis of the answers to the questionnaire “How much EASIER?”, and on the relevant monetary values.

#### Mean cost per administration procedure from the perspective of the patient and the society

Table 6 shows the direct healthcare costs, direct non-healthcare costs, and indirect costs per administration procedure.

**Table 6:** Direct healthcare costs, direct non-healthcare costs, and indirect costs per administration procedure

Cost type	Details	Mean cost/administration (€)		
		IV procedure	SC procedure	Delta
Direct healthcare costs	Direct healthcare costs	32.67	12.00	− 20.67
Indirect cost due to loss of patient productivity	Lost working time—at the expense of the patient	14.75	7.57	− 7.18
	Lost working time—social costs	42.19	21.65	− 20.54
	Unpaid activities—social costs	5.68	2.92	− 2.77
Indirect cost due to informal care	Cost borne by the society	31.10	15.96	− 15.14
Non-healthcare costs borne by the patient	Transport	30.68	30.68	–
	Babysitters and other paid professionals	2.38	1.22	− 1.16

In some cases, rounding prevents the sum to seem precise

Table 7 shows the impact of SC administration from the society, MS center, and patient perspectives.

**Table 7:** Impact of SC administration from the society, MS center, and patient perspectives

	IV administration (€)	SC administration (€)	Difference (€)	Difference (%)
Total cost in the perspective of society	159.47	92.00	− 67.46	− 42%
Total cost in the MS center perspective	32.67	12.00	− 20.67	− 63%
Total cost in the patient perspective	47.82	39.48	− 8.34	− 17%

In some cases, rounding prevents the sum to seem precise

About non-healthcare costs, when valorizing the transport costs, it should be taken into account that most patients reached the MS center by private vehicles, 11% by public transport, 2 patients took an airplane, and a minority of patients walked to the center. The mean transport cost per administration was € 30.68 (± 36.09) (Table 6), with a wide interindividual range, from € 0 (those who went on foot) to € 200 (those who traveled 400 km round trip by a private vehicle).

For those (9% of patients) who needed to pay personnel (e.g., baby-sitter) while away due to natalizumab administration, the disbursement was € 27.50 per administration (€ 2.38 when redistributing the costs over the total number of patients, Table 6).

Concerning indirect costs, it has to be noticed that the time spent to travel from home to the MS center and to stay there is an indirect cost for the society, since in this timespan the patient cannot carry out his normal activities, whether they are formally paid or not. From a financial point of view, three sub-indicators have been elaborated: the first is related to the loss of earnings by patients, the second is charged to the society as supported by the social security bodies, and the third is again borne by the society, which has to replace patients in various active roles, such as taking care of children, parents, domestic environment, or others through voluntary work.

This analysis showed that the time combination of travel + waiting + IV administration resulted on average in the loss of 3.61 and 3.47 h of work for male and female patients, respectively. This time was reimbursed by the social security bodies in 65–74% of workers. The unpaid time was estimated at about 2 h per administration. As reported in Table 6, the average cost for the lost working time at the expense of the patient was € 14.75 (± 29.98; range = € 0–130.31), the social cost for the lost working time was € 42.19 (± 61.45; range = € 0–431.25), and the social costs for unpaid activities was € 5.68 (± 8.94; range = € 0–81.72). Taking together, these costs add up to € 62.63 per administration.

As reported above, 48% of patients need the help of informal caregivers, 35% of whom have a paid job. Considering the average loss of hours declared by the patients, the mean cost for society (distributing the average on all the patients) was equal to € 31.10 per administration.

Overall, the total costs to be paid by the society was equal to € 159 for each IV administration. By applying the reduction in total patient time estimated in phase 2 of the study (Online Resource 5) to the time-dependent cost items, we estimated the reduction in the case of SC administration. As shown in Table 7, this saving was quantified as € 67.46 per administration from the perspective of the society as a whole, i.e., 42% of the total cost for IV administration borne by the society.

The direct financial burden on the patients and families was just over 8 euros (Table 7). In this case, the reduction percentage was consistently lower (less than 20%). This is easily explained by the absence of a differential effect of the route of administration, with the same setting, on transport costs, which represent the most relevant component of this type of cost in the patient’s perspective.

## Discussion

The EASIER study was designed and conducted to estimate the impact associated with the use of SC natalizumab vs. IV natalizumab in patients with RRMS from the perspectives of the MS center, the patient, and the society. The use of SC administration route, compared to the IV one, may reduce the consumption of healthcare resources, decrease the burden of therapy for patients and the time commitment for HCPs, as already demonstrated with other drugs in different therapeutic settings [25, 40–42].

Thanks to the measurements carried out in part 1 of the study, it was calculated that each IV procedure requires patients to spend on average two and a half hours in the MS center and HCPs to dedicate half an hour per infusion procedure. According to the questionnaires administered to HCPs and patients in part 2 and part 3, respectively, it was estimated that the SC administration route would result in a 50% and 55% reduction in patient time and HCPs active working time, respectively. As a consequence, a 63% cost reduction is estimated for the MS center per natalizumab administration procedure. In addition, indirect costs due to the loss of productivity of patients and caregivers would decrease as well as direct non-medical costs with cost reduction also from the point of view of the patient (− 17%) and society (− 42%).

In lifelong diseases, such as MS, the quality of life of patients should be carefully considered when evaluating the effects of a treatment. An Italian study carried out by Battaglia and colleagues [43] demonstrated that MS affected health-related QoL in all five domains evaluated by EQ-5D questionnaire, i.e., pain/discomfort, usual activities, mobility, anxiety/depression, and self-care, and that the severity and the domains involved changed with advancing disease. Another study in MS setting [44] used a different tool to evaluate the QoL, i.e., VAS, which is the same tool that we adopted in this study.

According to VAS in part 3 of the study, natalizumab administration was estimated to result in an important reduction in the perceived QoL in the days of IV infusion compared with days without infusion, regardless of long-lasting experience with infusions. When patients were asked to estimate the expected QoL in case of subcutaneous administration, their score was slightly higher than the intravenous one. This is in line with a study performed in oncology setting [28], which found that among patients who had the opportunity to test both routes of administration of another monoclonal antibody (trastuzumab), the subcutaneous route was preferred in 91.5% of patients. Among the advantages of SC administration, which may be particularly appreciated by patients, there are the elimination of the need for venous access and the possibility to use different injection sites [29]. Preliminary results of an observational study conducted in Germany on SC natalizumab [45] confirm this trend. In particular, at baseline, 89.6% (*n* = 163) of recruited MS patients preferred SC to IV natalizumab and 98.7% of them (*n* = 156) were satisfied with their choice. After 6 months of treatment, 96.4% of patients preferred SC natalizumab and 98.1% of them were satisfied with their choice. In addition, SC natalizumab was well tolerated; 8.4% (*n* = 14) patients reported adverse events related to natalizumab injection. The intensity of these events was mild or moderate. Furthermore, SC administration, with respect to IV infusion, lasted 1.5 h less, for both patients and HCPs.

In the EASIER study, working patients were more prone to give higher expected QoL scores for SC administration than non-working patients. It is worth considering that more than 70% of responders to the questionnaire “EASIER for you?” (part 3) had a paid job and that the time spent for monthly infusions (5 h per administration) was not completely reimbursed by welfare measures. In addition, the absences from work to receive natalizumab administration had an impact on other aspects of the working life, such as the need for reducing the working hours, the level of duties, up to stop working, although it is probably difficult to draw a line between the working problems directly related to multiple sclerosis and those related only to drug administration.

Similar to our study, in the project “Tieni al Tempo” [46], where ad-hoc questionnaires were administered to 255 Italian patients affected by MS with scheduled treatment sessions with a disease-modifying therapy (in general, monoclonal antibodies), the day of infusion was considered more stressful than the days without infusion by 80% of responders. In addition, 60% of responders declared that the quality of the infusion time was the main problem associated with the infusion in the MS center. Therefore, the reduction in the total patient time that was estimated in our study (152 min measured for IV infusion vs. 78 min estimated for SC administration) and confirmed in other setting evaluating the comparison IV vs. SC administrations [27] may result in an additional improvement in the quality of life perceived. In working patients, the reduction in the time spent in the MS center that may be achieved with subcutaneous administration also decreases the loss of productivity, as already calculated in other settings [28, 30]. In addition, caregivers also undergo loss of productivity, and this is particularly apparent when considering that in our study one-third of informal caregivers accompanying the patient to the MS center had a paid job.

The subcutaneous administration offers further advantages as compared to intravenous infusion. First, as already reported by other studies on IV vs. SC administrations [25, 26], the EASIER study estimated a decrease in the active time spent by HCPs for the administration, as more than a half of total time spent by HCPs is estimated to be spared (29 min measured for IV infusion vs. 13 min estimated for SC administration). This extra-time may be used by HCPs to deal with other tasks favoring the efficiency of the system. Second, the infusion chair, being occupied for a shorter time (125 min measured for IV infusion vs. 51 min estimated for SC administration), is free to host other patients, whether affected by MS or other pathologies, as reported by other studies on rituximab [25] and trastuzumab [26]. As a consequence, the waiting lists for these categories of patients should be shortened and the general management of the health administration improved, as demonstrated in other settings [24]. Finally, the decreased consumption of consumables such as infusion set and disposable gloves, also confirmed by studies evaluating the advantages of subcutaneous administration [24, 28, 29], far from resulting just in a decrease in costs (which are generally quite low), have also the advantage of reducing the waste quantity and, above all, the time spent for waste disposal, thus freeing further time for HCPs.

A reliable and detailed picture of the cost of illness in Italy comes from a recent study of Battaglia et al. [4], which took into account several items, when calculating direct healthcare costs (hospital admissions, rehabilitation at home, day hospital and outpatient visits at MS centers, additional outpatient medical visits, tests and diagnostic procedures, pharmacological treatments, and external technical aids/orthoses), direct non-healthcare costs (transport, paid assistance, house and car modifications due to MS), and indirect costs (patients and caregivers’ productivity losses due to MS). The mean annual cost per patient varied according with disease severity, i.e., € 29,676, € 43,464, and € 53,454 for mild, moderate, and severe MS, respectively.

The EASIER study may be compared with a Spanish study that estimated the time spent for natalizumab IV infusion and SC administration and the relevant costs (not considering natalizumab acquisition costs), based on the judgement of a dedicated task force that provided data based on experience on IV administration and estimations on SC administrations (recently authorized in Spain for natalizumab) [47]. The total time spent per patient in 2 years, including administration time and time spent by patients and caregivers, was 55–61% lower with subcutaneous administration with respect to IV infusion. Similar time reductions were estimated in the EASIER study, as the total patient time, total infusion chair time, and total HCP time decreased by 48%, 59%, and 55%, respectively. Concerning costs, the Spanish study estimated a 66–70% cost reduction in SC administration when compared with IV administration in a 2-year time [47]. These costs included administration process and the productivity loss for patients and caregivers. A direct comparison between the Spanish and the present study shows the following results: reduction for administration costs 76% vs 63%, decrease in productivity loss for patients 45–55% vs 49% and decrease in productivity loss for caregivers 69–74% vs 49%.

In the EASIER study, the economic quantification of the data collected on IV infusions allowed the estimation of the main outcomes of the study and indicates that the average total healthcare cost borne by the MS center per administration procedure may be reduced by about two-thirds (− 63%), thanks to the SC administration. From the society perspective, including direct non-healthcare costs and indirect costs as well as direct healthcare costs, the current average cost per single administration would be reduced by approximately 42%.

From the patient’s perspective, although the cost of transport to the MS center remains unchanged, a reduction in costs of approximately 17% was also estimated, due to the decrease in the time spent in the health care facility, resulting in a reduction in the working hours lost and the hours spent as formal care by accompanying family members and friends during the administration.

The main limitation of this study is that the impact of subcutaneous administration of natalizumab on costs, work, and QoL was estimated by HCPs and patients using questionnaires, instead of being measured during clinical practice. However, at present, since SC administration of natalizumab is not yet authorized in Italy, a measurement is not possible. Therefore, it will be necessary to confirm the results of this study with real-world data also for subcutaneous natalizumab.

In conclusion, this study confirms data coming from other therapeutic settings, as it estimates a reduction of the impact of SC vs. IV administration in terms of time, costs, and burden for the patients. Only focusing on aspects affecting healthcare economics, SC route, if compared with IV route for natalizumab administration, is expected to save 78 min of the total current 152 min procedure time, resulting in a reduction in the time spent by the patient in the hospital; active HCP working time per procedure is estimated to drop from 29 to 13 min. Coupled with a slight reduction in material consumption, time savings are expected to positively impact on the total costs per procedure, with a reduction of about 42%, 63%, and 17% (− 68 €, − 21 €, and − 8 €) from the perspectives of the society, MS center, and patient, respectively. In addition, a positive impact on the organization and efficiency of the MS center is expected, as HCPs would have further time to perform other tasks and the infusion chair may be used by an increased number of patients, thus reducing the waiting lists and then favoring the efficiency of the system. We also confirm that the SC route is widely preferred by patients over the IV one, including those affected by MS.

### Supplementary Information

Below is the link to the electronic supplementary material.

Supplementary file1 (PDF 135 KB)

Supplementary file2 (PDF 111 KB)

Supplementary file3 (PDF 207 KB)

Supplementary file4 (PDF 196 KB)

Supplementary file5 (PDF 113 KB)

Supplementary file6 (PDF 116 KB)

Supplementary file7 (PDF 111 KB)

Supplementary file8 (PDF 160 KB)

## Data Availability

The datasets generated during and/or analyzed during the current study are available from the corresponding author on reasonable request.

## References

[1] Atlas of MS 2020—Epidemiology report. MS International Federation. https://www.msif.org/resource/atlas-of-ms-2020/. Accessed 1 Dec 2022

[2] Number of people with MS | Atlas of MS. https://www.atlasofms.org/map/italy/epidemiology/number-of-people-with-ms. Accessed 1 Dec 2022

[3] Olesen J, Gustavsson A, Svensson M (2012). The economic cost of brain disorders in Europe. Eur J Neurol.

[4] Battaglia MA, Bezzini D, Cecchini I (2022). Patients with multiple sclerosis: a burden and cost of illness study. J Neurol.

[5] European Medicines Agency. Summary of product characteristics. Tysabri. https://www.ema.europa.eu/en/documents/product-information/tysabri-epar-product-information_en.pdftysabri-epar-product-information_en.pdf. Accessed 1 Dec 2022

[6] Gazzetta Ufficiale. https://www.gazzettaufficiale.it/atto/serie_generale/caricaDettaglioAtto/originario?atto.dataPubblicazioneGazzetta=2020-11-27&atto.codiceRedazionale=20A06441&elenco30giorni=false. Accessed 14 Dec 2022

[7] Polman CH, O’Connor PW, Havrdova E (2006). A randomized, placebo-controlled trial of natalizumab for relapsing multiple sclerosis. N Engl J Med.

[8] Jacobsen C, Hagemeier J, Myhr K-M (2014). Brain atrophy and disability progression in multiple sclerosis patients: a 10-year follow-up study. J Neurol Neurosurg Psychiatry.

[9] Prosperini L, De Angelis F, De Angelis R (2015). Sustained disability improvement is associated with T1 lesion volume shrinkage in natalizumab-treated patients with multiple sclerosis. J Neurol Neurosurg Psychiatry.

[10] Rinaldi F, Calabrese M, Seppi D (2012). Natalizumab strongly suppresses cortical pathology in relapsing–remitting multiple sclerosis. Mult Scler.

[11] Zivadinov R, Dwyer M, Hussein S (2012). Voxel-wise magnetization transfer imaging study of effects of natalizumab and IFNβ-1a in multiple sclerosis. Mult Scler.

[12] Mattioli F, Stampatori C, Bellomi F (2015). Natalizumab significantly improves cognitive impairment over three years in MS: pattern of disability progression and preliminary MRI findings. PLoS ONE.

[13] Morrow SA, O’Connor PW, Polman CH (2010). Evaluation of the symbol digit modalities test (SDMT) and MS neuropsychological screening questionnaire (MSNQ) in natalizumab-treated MS patients over 48 weeks. Mult Scler.

[14] Svenningsson A, Falk E, Celius EG (2013). Natalizumab treatment reduces fatigue in multiple sclerosis. Results from the TYNERGY trial; a study in the real life setting. PLoS ONE.

[15] Wilken J, Kane RL, Sullivan CL (2013). Changes in fatigue and cognition in patients with relapsing forms of multiple sclerosis treated with natalizumab: the ENER-G study. Int J MS Care.

[16] Kunkel A, Fischer M, Faiss J (2015). Impact of natalizumab treatment on fatigue, mood, and aspects of cognition in relapsing-remitting multiple sclerosis. Front Neurol.

[17] Rudick RA, Miller D, Hass S (2007). Health-related quality of life in multiple sclerosis: effects of natalizumab. Ann Neurol.

[18] Yildiz M, Tettenborn B, Putzki N (2011). Multiple sclerosis-associated fatigue during disease-modifying treatment with natalizumab, interferon-beta and glatiramer acetate. Eur Neurol.

[19] Wickström A, Nyström J, Svenningsson A (2013). Improved ability to work after one year of natalizumab treatment in multiple sclerosis. Analysis of disease-specific and work-related factors that influence the effect of treatment. Mult Scler.

[20] Olofsson S, Wickström A, Häger Glenngård A (2011). Effect of treatment with natalizumab on ability to work in people with multiple sclerosis: productivity gain based on direct measurement of work capacity before and after 1 year of treatment. BioDrugs.

[21] Wickström A, Dahle C, Vrethem M, Svenningsson A (2014). Reduced sick leave in multiple sclerosis after one year of natalizumab treatment. A prospective ad hoc analysis of the TYNERGY trial. Mult Scler.

[22] Plavina T, Fox EJ, Lucas N (2016). A randomized trial evaluating various administration routes of natalizumab in multiple sclerosis. J Clin Pharmacol.

[23] Trojano M, Ramió-Torrentà L, Grimaldi LM (2021). A randomized study of natalizumab dosing regimens for relapsing-remitting multiple sclerosis. Mult Scler.

[24] Dent S, Ammendolea C, Christofides A (2019). A multidisciplinary perspective on the subcutaneous administration of trastuzumab in HER2-positive breast cancer. Curr Oncol.

[25] De Cock E, Kritikou P, Sandoval M (2016). Time savings with rituximab subcutaneous injection versus rituximab intravenous infusion: a time and motion study in eight countries. PLoS ONE.

[26] De Cock E, Pivot X, Hauser N (2016). A time and motion study of subcutaneous versus intravenous trastuzumab in patients with HER2-positive early breast cancer. Cancer Med.

[27] Olofsson S, Norrlid H, Karlsson E (2016). Societal cost of subcutaneous and intravenous trastuzumab for HER2-positive breast cancer—an observational study prospectively recording resource utilization in a Swedish healthcare setting. Breast.

[28] Pivot X, Gligorov J, Müller V (2013). Preference for subcutaneous or intravenous administration of trastuzumab in patients with HER2-positive early breast cancer (PrefHer): an open-label randomised study. Lancet Oncol.

[29] Dychter SS, Gold DA, Haller MF (2012). Subcutaneous drug delivery: a route to increased safety, patient satisfaction, and reduced costs. J Infus Nurs.

[30] Lopez-Vivanco G, Salvador J, Diez R (2017). Cost minimization analysis of treatment with intravenous or subcutaneous trastuzumab in patients with HER2-positive breast cancer in Spain. Clin Transl Oncol.

[31] ISTAT (2020) L’occupazione nella sanità pubblica. 6 maggio. https://www.istat.it/it/files//2020/05/Statistica_today_sanit%C3%A0_2018.pdf. Accessed 17 Nov 2022

[32] Schivazappa C, Baldini E, Cortesi E (2007). Cost analysis of tumor-induced osteolysis treated with intravenous zoledronic acid and pamidronate: a time-motion study. Recenti Prog Med.

[33] Cicchetti A, Coretti S, Mascia D, et al (2018) Assessing social and economic impact of subcutaneous mAbs in oncology. Glob Reg Health Technol Assess. 10.33393/grhta.2018.443

[34] ACI (2022) Costi chilometrici. http://www.aci.it/i-servizi/servizi-online/costi-chilometrici.html. Accessed 17 Nov 2022

[35] Avenali A, Catalano G, Gregori M, Matteucci G (2020). Rail versus bus local public transport services: a social cost comparison methodology. Transport Res Interdiscip Perspect.

[36] Servizio taxi e tariffe applicate. Comune di Milano. https://www.comune.milano.it/aree-tematiche/mobilita/taxi. Accessed 17 Nov 2022

[37] Ebilcoba. Nuovo contratto collettivo nazionale di lavoro colf e badanti. https://www.inps.it/docallegatiNP/Mig/Allegati/701Nuovo_CCNL_colf_badanti_Ebilcoba.pdf. Accessed 17 Nov 2022

[38] ISTAT (2019) Multiscopo sulle famiglie: uso del tempo—file per la ricerca. https://www.istat.it/it/archivio/202520. Accessed 23 Nov 2022

[39] CNEL, ISTAT (2011) La valorizzazione economica del lavoro volontario nel settore non profit. https://www.redattoresociale.it/media/la_valorizzazione_economica_del_lavoro_volontario_nel_settore_non_profit_. Accessed 23 Nov 2022

[40] Rule S, Collins GP, Samanta K (2014). Subcutaneous vs intravenous rituximab in patients with non-Hodgkin lymphoma: a time and motion study in the United Kingdom. J Med Econ.

[41] Ponzetti C, Canciani M, Farina M (2016). Potential resource and cost saving analysis of subcutaneous versus intravenous administration for rituximab in non-Hodgkin’s lymphoma and for trastuzumab in breast cancer in 17 Italian hospitals based on a systematic survey. Clinicoecon Outcomes Res.

[42] Anderson KC, Landgren O, Arend RC (2019). Humanistic and economic impact of subcutaneous versus intravenous administration of oncology biologics. Future Oncol.

[43] Battaglia M, Kobelt G, Ponzio M (2017). New insights into the burden and costs of multiple sclerosis in Europe: results for Italy. Mult Scler.

[44] Kobelt G, Langdon D, Jönsson L (2019). The effect of self-assessed fatigue and subjective cognitive impairment on work capacity: the case of multiple sclerosis. Mult Scler.

[45] Gold R, Schmidt S, Motte J et al (2022) SISTER—subcutaneous: non-interventional, observational, prospective, German multicenter, open label study over 12 months for Tysabri Patient Preference. In: 38th Congress of the European Committee for treatment and research in multiple sclerosis (ECTRIMS 2022) 26th–28th October, 2022 | Amsterdam, Netherlands

[46] AISM, Biogen. Barometro della sclerosi multipla 2021. Tieni al Tempo—Vissuto e opinioni delle persone con sclerosi multipla sul valore del tempo dell’infusione di farmaci. https://www.aism.it/sites/default/files/Tieni_al_tempo.PDF. Accessed 19 Dec 2022

[47] Alonso AM, Arévalo AG, Becerril N et al (2022) Cost-analysis of subcutaneous versus intravenous administration for natalizumab in Multiple Sclerosis in Spain. In: European Academy of Neurology—8th Congress | 25–28 June 2022 EPO-393. https://biogenlinc-assets-bucket.s3.eu-central-1.amazonaws.com/04_Value%20of%20Time%20_%20EAN2022%20Poster%20Spain.pdf. Accessed 19 Dec 2022

