# Supplementary material for: Intravenous or subcutaneous natalizumab in patients with relapsing–remitting multiple sclerosis: investigation on efficiency and savings—the EASIER study
Source: J Neurol. 2023 Sep 16;271(1):340–54. doi: 10.1007/s00415-023-11955-0 (PMC10769988; doi:10.1007/s00415-023-11955-0)
Supplement: Supplementary file 1 — Supplementary file1 (PDF 135 KB) [file 415_2023_11955_MOESM1_ESM.pdf]

Journal of Neurology

Intravenous or Subcutaneous Natalizumab in Patients with Relapsing Remitting Multiple Sclerosis: Investigation on Efficiency and Savings—The EASIER Study

Massimo Filippi[1,2], Luigi Grimaldi[3], Antonella Conte[4,5,6], Rocco Totaro[7], Maria Rosaria Valente[8], Simona Malucchi[9], Franco Granella[10], Cinzia Cordioli[11], Vincenzo Brescia Morra[12], Chiara Zanetta[1], Daria Perini[13], Laura Santoni[13]; on behalf of the EASIER study working group

[1]Neurology Unit, Neurorehabilitation Unit, Neurophysiology Service, and Neuroimaging Research Unit, Division of Neuroscience, IRCCS San Raffaele Scientific Institute, Milan, Italy; [2]Vita-Salute San Raffaele University, Milan, Italy; [3]Multiple Sclerosis Center, Fondazione Istituto G. Giglio, Cefalù (PA), Italy; [4]Department of Human Neurosciences, Sapienza, University of Rome, Italy; [5]Multiple Sclerosis Center Policlinico Umberto I Hospital, Rome, Italy; [6]IRCCS Neuromed, Pozzilli (IS), Italy; [7]Demyelinating Disease Center, Department of Neurology, San Salvatore Hospital, L'Aquila, Italy; [8]Clinical Neurology, Santa Maria della Misericordia University Hospital and Department of Medicine, University of Udine, Udine, Italy; [9]SCDO Neurologia, S. Luigi Gonzaga University Hospital, Orbassano (TO), Italy; [10]Department of Medicine and Surgery, University Hospital of Parma, Parma, Italy; [11]Multiple Sclerosis Center, ASST Spedali Civili di Brescia, Montichiari Hospital (Brescia), Italy; [12]Multiple Sclerosis Clinical Care and Research Center, Federico II University Hospital—Department of Neuroscience (NSRO), Naples, Italy; [13]Biogen Italia, Milan, Italy
Corresponding author: Massimo Filippi, filippi.massimo@hsr.it

Tasks	Description	Patient time	HCP time	Chair time	Pre - infusion	Post-infusion
1 – Material preparation	Material pick-up, except the drug, placement next to the infusion chair		X		X	
2 – Drug pick-up at the H pharmacy	Round trip to the hospital pharmacy, pick-up of the drug, placement in the ward refrigerator		X		X	
3 – Patient preparation	Undressing-dressing, parameters measurement (temperature, blood pressure, heart rate, etc.), possible visit, seating on chair/bed, possible pre-dressing, disinfection, and venous access	X	X	X	X	
4 – Drug preparation	Taking, dilution, visual inspection	X	X	X	X	
5 – Drug infusion	Total infusion length	X		X		
5.1 – Drug infusion—Active time*	Infusion set attachment. Outflow connection to the catheter needle, other active time dedicated to the patient during the infusion for routine control or upon request		X			
6 – Post-infusion tasks	Intravenous flushing, set removal, dressing, clearing of infusion chair/bed (if monitoring elsewhere)	X	X	X		X
7 – Monitoring	The total time between post-infusion steps and when the patient leaves the MS unit	X		X		X
7.1 – Monitoring—Active Time*	Accommodating the patient in the monitoring site (if different from the infusion site), additional active time dedicated to the patient during monitoring for routine check-ups or upon request, patient discharge		X			X
8 – Infusion field cleaning and waste disposal	Cleaning of the infusion chair/bed, waste disposal, relocation of unused material. Please note: write down the armchair/bed among the materials used if unusable by other patients during cleaning		X	X		X

Online Resource 1. Splitting of the time spent for the procedure.

* 5.1 e 7.1 activities, while being part of the patient time, are not added because already included in 5 and 7 activities, respectively.

H = hospital; HCP = healthcare professional
