# Supplementary material for: Intravenous or subcutaneous natalizumab in patients with relapsing–remitting multiple sclerosis: investigation on efficiency and savings—the EASIER study
Source: J Neurol. 2023 Sep 16;271(1):340–54. doi: 10.1007/s00415-023-11955-0 (PMC10769988; doi:10.1007/s00415-023-11955-0)
Supplement: Supplementary file 2 — Supplementary file2 (PDF 111 KB) [file 415_2023_11955_MOESM2_ESM.pdf]

Journal of Neurology

Intravenous or Subcutaneous Natalizumab in Patients with Relapsing Remitting Multiple Sclerosis: Investigation on Efficiency and Savings—The EASIER Study

Massimo Filippi[1,2], Luigi Grimaldi[3], Antonella Conte[4,5,6], Rocco Totaro[7], Maria Rosaria Valente[8], Simona Malucchi[9], Franco Granella[10], Cinzia Cordioli[11], Vincenzo Brescia Morra[12], Chiara Zanetta[1], Daria Perini[13], Laura Santoni[13]; on behalf of the EASIER study working group

[1]Neurology Unit, Neurorehabilitation Unit, Neurophysiology Service, and Neuroimaging Research Unit, Division of Neuroscience, IRCCS San Raffaele Scientific Institute, Milan, Italy; [2]Vita-Salute San Raffaele University, Milan, Italy; [3]Multiple Sclerosis Center, Fondazione Istituto G. Giglio, Cefalù (PA), Italy; [4]Department of Human Neurosciences, Sapienza, University of Rome, Italy; [5]Multiple Sclerosis Center Policlinico Umberto I Hospital, Rome, Italy; [6]IRCCS Neuromed, Pozzilli (IS), Italy; [7]Demyelinating Disease Center, Department of Neurology, San Salvatore Hospital, L'Aquila, Italy; [8]Clinical Neurology, Santa Maria della Misericordia University Hospital and Department of Medicine, University of Udine, Udine, Italy; [9]SCDO Neurologia, S. Luigi Gonzaga University Hospital, Orbassano (TO), Italy; [10]Department of Medicine and Surgery, University Hospital of Parma, Parma, Italy; [11]Multiple Sclerosis Center, ASST Spedali Civili di Brescia, Montichiari Hospital (Brescia), Italy; [12]Multiple Sclerosis Clinical Care and Research Center, Federico II University Hospital—Department of Neuroscience (NSRO), Naples, Italy; [13]Biogen Italia, Milan, Italy
Corresponding author: Massimo Filippi, filippi.massimo@hsr.it

Subjects/jobs	Male (€)	Female (€)	Source
14-19 y	11.61	11.17	ISTAT 2018 [1], updated 2021
20-29 y	13.54	12.95	
30-39 y	17.25	16.20	
40-49 y	20.58	18.77	
50-59 y	23.79	21.68	
60+ y	29.52	24.82	
Upaid lost hour	2.31	3.25	Uso del tempo 2013-2014 [1], spread over 16 hours/day
Caretaker	7.84		Mean between super B level for autonomous patients and super C/D for non-autonomous patients [2]
Maid/baby-sitter	7.16		Mean between super B level (baby-sitter) and B for housework [2]
Volunteer	12.61		Rapporto CNEL-ISTAT [3] updated 2021

Online Resource 2. Time value: gross hourly wage.

References

1. ISTAT (2019) Multiscopo sulle famiglie: uso del tempo - file per la ricerca. https://www.istat.it/it/archivio/202520. Accessed 23 Nov 2022

2. Ebilcoba. Nuovo contratto collettivo nazionale di lavoro colf e badanti. https://www.inps.it/docallegatiNP/Mig/Allegati/701Nuovo_CCNL_colf_badanti_Ebilcoba.pdf. Accessed 17 Nov 2022

3. CNEL, ISTAT (2011). La valorizzazione economica del lavoro volontario nel settore non profit. https://www.redattoresociale.it/media/la_valorizzazione_economica_del_lavoro_volontario_nel_settore_non_profit_ Accessed 23 Nov 2022
