# Supplementary material for: Intravenous or subcutaneous natalizumab in patients with relapsing–remitting multiple sclerosis: investigation on efficiency and savings—the EASIER study
Source: J Neurol. 2023 Sep 16;271(1):340–54. doi: 10.1007/s00415-023-11955-0 (PMC10769988; doi:10.1007/s00415-023-11955-0)
Supplement: Supplementary file 5 — Supplementary file5 (PDF 113 KB) [file 415_2023_11955_MOESM5_ESM.pdf]

Journal of Neurology

Intravenous or Subcutaneous Natalizumab in Patients with Relapsing Remitting Multiple Sclerosis: Investigation on Efficiency and Savings—The EASIER Study

Massimo Filippi[1,2], Luigi Grimaldi[3], Antonella Conte[4,5,6], Rocco Totaro[7], Maria Rosaria Valente[8], Simona Malucchi[9], Franco Granella[10], Cinzia Cordioli[11], Vincenzo Brescia Morra[12], Chiara Zanetta[1], Daria Perini[13], Laura Santoni[13]; on behalf of the EASIER study working group

[1]Neurology Unit, Neurorehabilitation Unit, Neurophysiology Service, and Neuroimaging Research Unit, Division of Neuroscience, IRCCS San Raffaele Scientific Institute, Milan, Italy; [2]Vita-Salute San Raffaele University, Milan, Italy; [3]Multiple Sclerosis Center, Fondazione Istituto G. Giglio, Cefalù (PA), Italy; [4]Department of Human Neurosciences, Sapienza, University of Rome, Italy; [5]Multiple Sclerosis Center Policlinico Umberto I Hospital, Rome, Italy; [6]IRCCS Neuromed, Pozzilli (IS), Italy; [7]Demyelinating Disease Center, Department of Neurology, San Salvatore Hospital, L'Aquila, Italy; [8]Clinical Neurology, Santa Maria della Misericordia University Hospital and Department of Medicine, University of Udine, Udine, Italy; [9]SCDO Neurologia, S. Luigi Gonzaga University Hospital, Orbassano (TO), Italy; [10]Department of Medicine and Surgery, University Hospital of Parma, Parma, Italy; [11]Multiple Sclerosis Center, ASST Spedali Civili di Brescia, Montichiari Hospital (Brescia), Italy; [12]Multiple Sclerosis Clinical Care and Research Center, Federico II University Hospital—Department of Neuroscience (NSRO), Naples, Italy; [13]Biogen Italia, Milan, Italy
Email address of the corresponding author

**Tasks**	**Unchanged** (n. responding HCP)	**Changed** (n. responding HCP)	**Null** (n. responding HCP)	**Delta** (minutes)
1 – Material preparation	6	20	0	-0.54
2 – Drug pick-up at the H pharmacy	24	2	0	0
3 – Patient preparation	5	20	0	-4.84
4 – Drug preparation*	NA	NA	NA	-2.31
5 – Drug infusion	0	25	0	-45.84
5.1 – Drug infusion—Active time**	9	14	2	-2.22
6 – Post-infusion tasks**	1	20	4	-5.39
7 – Monitoring	10	15	0	-14.8
7.1 – Monitoring—Active Time***	13	10	2	0
8 – Infusion field cleaning and waste disposal	1	23	1	-0.84

Online Resource 5. How much EASIER? Impact estimations on every task according to the responders.

HCP = healthcare professional

* IV-specific task, and as such omitted from questionnaire—the delta corresponds to mean observed value for IV, that would be saved with SC as not pertinent

** Estimated time savings (-4.3 min for task 5.1 and -6.1 min for task 6) greater than observed mean value for IV: delta limited to mean IV time as logic constraint

*** Set at 0, as majority of HCP responders answered it would be unchanged, despite mean estimated saving resulted in 2.97 minutes
