# Supplementary material for: Intravenous or subcutaneous natalizumab in patients with relapsing–remitting multiple sclerosis: investigation on efficiency and savings—the EASIER study
Source: J Neurol. 2023 Sep 16;271(1):340–54. doi: 10.1007/s00415-023-11955-0 (PMC10769988; doi:10.1007/s00415-023-11955-0)
Supplement: Supplementary file 6 — Supplementary file6 (PDF 116 KB) [file 415_2023_11955_MOESM6_ESM.pdf]

Journal of Neurology

Intravenous or Subcutaneous Natalizumab in Patients with Relapsing Remitting Multiple Sclerosis: Investigation on Efficiency and Savings—The EASIER Study

Massimo Filippi[1,2], Luigi Grimaldi[3], Antonella Conte[4,5,6], Rocco Totaro[7], Maria Rosaria Valente[8], Simona Malucchi[9], Franco Granella[10], Cinzia Cordioli[11], Vincenzo Brescia Morra[12], Chiara Zanetta[1], Daria Perini[13], Laura Santoni[13]; on behalf of the EASIER study working group

[1]Neurology Unit, Neurorehabilitation Unit, Neurophysiology Service, and Neuroimaging Research Unit, Division of Neuroscience, IRCCS San Raffaele Scientific Institute, Milan, Italy; [2]Vita-Salute San Raffaele University, Milan, Italy; [3]Multiple Sclerosis Center, Fondazione Istituto G. Giglio, Cefalù (PA), Italy; [4]Department of Human Neurosciences, Sapienza, University of Rome, Italy; [5]Multiple Sclerosis Center Policlinico Umberto I Hospital, Rome, Italy; [6]IRCCS Neuromed, Pozzilli (IS), Italy; [7]Demyelinating Disease Center, Department of Neurology, San Salvatore Hospital, L'Aquila, Italy; [8]Clinical Neurology, Santa Maria della Misericordia University Hospital and Department of Medicine, University of Udine, Udine, Italy; [9]SCDO Neurologia, S. Luigi Gonzaga University Hospital, Orbassano (TO), Italy; [10]Department of Medicine and Surgery, University Hospital of Parma, Parma, Italy; [11]Multiple Sclerosis Center, ASST Spedali Civili di Brescia, Montichiari Hospital (Brescia), Italy; [12]Multiple Sclerosis Clinical Care and Research Center, Federico II University Hospital—Department of Neuroscience (NSRO), Naples, Italy; [13]Biogen Italia, Milan, Italy
Corresponding author: Massimo Filippi, filippi.massimo@hsr.it

	**N. responders (%)**	**Mean (SD)**	**Range**
Round trip distance (km traveled)	273 (92%)	73.6 (159.4)	1.5–2400
Travel time (hours)	272 (92%)	1.6 (1.4)	0–10
Means of transport	290 (98%)		
• Private vehicle	249 (86%)		
• Public transport	31 (11%)		
• Taxi	5 (2%)		
• Other	5 (2%)*		

Online Resource 6. Time and means of transport for IV procedure.

*3 on foot and 2 by plane
