# Supplementary material for: Intravenous or subcutaneous natalizumab in patients with relapsing–remitting multiple sclerosis: investigation on efficiency and savings—the EASIER study
Source: J Neurol. 2023 Sep 16;271(1):340–54. doi: 10.1007/s00415-023-11955-0 (PMC10769988; doi:10.1007/s00415-023-11955-0)
Supplement: Supplementary file 8 — Supplementary file8 (PDF 160 KB) [file 415_2023_11955_MOESM8_ESM.pdf]

Journal of Neurology

Intravenous or Subcutaneous Natalizumab in Patients with Relapsing Remitting Multiple Sclerosis: Investigation on Efficiency and Savings—The EASIER Study

Massimo Filippi[1,2], Luigi Grimaldi[3], Antonella Conte[4,5,6], Rocco Totaro[7], Maria Rosaria Valente[8], Simona Malucchi[9], Franco Granella[10], Cinzia Cordioli[11], Vincenzo Brescia Morra[12], Chiara Zanetta[1], Daria Perini[13], Laura Santoni[13]; on behalf of the EASIER study working group

[1]Neurology Unit, Neurorehabilitation Unit, Neurophysiology Service, and Neuroimaging Research Unit, Division of Neuroscience, IRCCS San Raffaele Scientific Institute, Milan, Italy; [2]Vita-Salute San Raffaele University, Milan, Italy; [3]Multiple Sclerosis Center, Fondazione Istituto G. Giglio, Cefalù (PA), Italy; [4]Department of Human Neurosciences, Sapienza, University of Rome, Italy; [5]Multiple Sclerosis Center Policlinico Umberto I Hospital, Rome, Italy; [6]IRCCS Neuromed, Pozzilli (IS), Italy; [7]Demyelinating Disease Center, Department of Neurology, San Salvatore Hospital, L'Aquila, Italy; [8]Clinical Neurology, Santa Maria della Misericordia University Hospital and Department of Medicine, University of Udine, Udine, Italy; [9]SCDO Neurologia, S. Luigi Gonzaga University Hospital, Orbassano (TO), Italy; [10]Department of Medicine and Surgery, University Hospital of Parma, Parma, Italy; [11]Multiple Sclerosis Center, ASST Spedali Civili di Brescia, Montichiari Hospital (Brescia), Italy; [12]Multiple Sclerosis Clinical Care and Research Center, Federico II University Hospital—Department of Neuroscience (NSRO), Naples, Italy; [13]Biogen Italia, Milan, Italy
Corresponding author: Massimo Filippi, filippi.massimo@hsr.it

Integrative and exploratory analyses on patient preferences about subcutaneous administration

A series of exploratory analyses was carried out on the values assigned by patients to subcutaneous (SC) administration aiming at evaluating possible determinants, in particular the working status and the previous experience in the use of subcutaneous treatments.

First, the preference values assigned by patients with vs. without paid work were directly compared. Similarly, the values assigned by patients never vs. previously treated with SC treatments were directly compared. Wilcoxon non-parametric test was used to evaluate the difference between groups.

Working status was strongly and positively associated with the value assigned to a possible day with SC administration (Online Resource 8-Supplementary Table 1).

	Mean value±SD (N=237)	Group	Mean value±SD	p-value
Working status	68.9±23.0	Without paid work (N=61)	61.5±25.3	0.0104
		With paid work (N=174)	71.2±21.7	
Previous experience in the use of SC	68.9±23.0	Never treated with SC treatments (N=143)	70.4±23.4	0.1403
		Previously treated with SC treatments (N=94)	66.5±22.3	

Online Resource 8-Supplementary Table 1. Preferences for the subcutaneous administration based on the working status and the previous experience in the use of subcutaneous treatments.

SC = subcutaneous; SD = standard deviation

The values were slightly higher in patients never treated with SC treatments with respect to patients previously treated with SC treatments, but without reaching the statistical significance (Online Resource 8-Supplementary Table 1).

The impact of the abovementioned factors was further investigated by means of multivariate regression analysis, that evaluates the association between measured factors and the different preference expressed by patients on the administration routes, while simultaneously adjusting for the assessment on the quality of life in days without administrations and other possible confounders.

These analyses are of incremental nature, i.e., instead of focusing on the absolute values assigned by patients to the different administration routes, they analyze the difference between scores assigned to each pair of administration routes. As a consequence, the examined sample does not correspond to the total sample, as it includes just patients having assigned a value to the routes of administration analyzed.

The mean overall differences between assigned preferences are summarized in Online Resource 8-Supplementary Table 2.

	IV	SC
Day with no administration	-12.2	-8.4
Day with IV administration		+2.3

Online Resource 8-Supplementary Table 2. Mean differences between estimated quality of life. between IV and SC administration of natalizumab are shown: row vs. column

IV = intravenous; SC = subcutaneous

The results of the multivariate analyses conducted both considering the previous experience with SC treatments as dichotomous variable (“previous experience with SC treatments” yes vs. no— Online Resource 8-Supplementary Table 3) and as continuous variable (“months of therapy with SC treatments”— Online Resource 8-Supplementary Table 4) confirm a significant effect of working status.

Variables	Mean difference (95% CI)	p-value
Experience with SC treatments (yes vs. no)	-0.69 (-6.10 to 4.73)	0.804
Age (years)	0.15 (-0.13 to 0.42)	0.289
Sex (F vs. M)	-3.71 (-9.57 to 2.16)	0.215
Treatment with IV natalizumab (months)	-0.01 (-0.09 to 0.07)	0.765
**Currently working (yes vs. no)**	**6.91 (0.91 to 12.92)**	**0.024**
Accompanying person in the hospital (yes vs. no)	3.30 (-2.34 to 8.95)	0.252
**Value of the day without administration**	**0.21 (0.07 to 0.34)**	**0.003**
Value of the day with IV administration	**0.45 (0.32 to 0.58)**	**<0.001**

Online Resource 8-Supplementary Table 3. Multivariate regression analysis on patient preferences expressing the value assigned to a day with SC administration of natalizumab (experience with SC treatments as dichotomous variable)

Variables	Mean difference (95% CI)	p-value
Experience with SC treatments (months)	-0.05 (-0.13 to 0.02)	0.180
Age (years)	0.19 (-0.09 to 0.48)	0.183
Sex (F vs. M)	-3.57 (-9.41 to 2.27)	0.231
Treatment with IV natalizumab (months)	-0.01 (-0.09 to 0.07)	0.763
**Currently working (yes vs. no)**	**7.49 (1.46 to 13.51)**	**0.015**
Accompanying person in the hospital (yes vs. no)	4.21 (-1.65 to 10.07)	0.159
**Value of the day without administration**	**0.20 (0.06 to 0.33)**	**0.005**
Value of the day with IV administration	**0.46 (0.33 to 0.60)**	**0.001**

Online Resource 8-Supplementary Table 4. Multivariate regression analysis on patient preferences expressing the value assigned to a day with SC administration of natalizumab (experience with SC treatments as continuous variable)

Concerning the effect of previous SC treatments, there is a nonsignificant trend towards a reduced preference for SC natalizumab in patients with experience of SC treatments.
